# Healthcare system inputs and patient-reported outcomes: a study in adults with congenital heart defect from 15 countries

**DOI:** 10.1186/s12913-020-05361-9

**Published:** 2020-06-03

**Authors:** Liesbet Van Bulck, Eva Goossens, Koen Luyckx, Silke Apers, Erwin Oechslin, Corina Thomet, Werner Budts, Junko Enomoto, Maayke A. Sluman, Chun-Wei Lu, Jamie L. Jackson, Paul Khairy, Stephen C. Cook, Shanthi Chidambarathanu, Luis Alday, Katrine Eriksen, Mikael Dellborg, Malin Berghammer, Bengt Johansson, Andrew S. Mackie, Samuel Menahem, Maryanne Caruana, Gruschen Veldtman, Alexandra Soufi, Susan M. Fernandes, Kamila White, Edward Callus, Shelby Kutty, Philip Moons, on behalf of the APPROACH-IS consortium and the International Society for Adult Congenital Heart Disease (ISACHD)

**Affiliations:** 1 KU Leuven Department of Public Health and Primary Care, KU Leuven - University of Leuven, Kapucijnenvoer 35, Box 7001, B-3000 Leuven, Belgium; 2 Research Foundation Flanders (FWO), Brussels, Belgium; 3 Division of Nursing and Midwifery, Faculty of Medicine and Health Sciences, Centre for Research and Innovation in Care, University of Antwerp, Antwerp, Belgium; 4 KU Leuven School Psychology and Development in Context, KU Leuven – University of Leuven, Leuven, Belgium; 5 UNIBS, University of the Free State, Bloemfontein, South Africa; 6 Department of Gynaecology and Obstetrics, University Hospitals Leuven, Leuven, Belgium; 7 Toronto Congenital Cardiac Centre for Adults, Peter Munk Cardiac Centre, University Health Network, University of Toronto, Toronto, Canada; 8 Center for Congenital Heart Disease, Inselspital - Bern University Hospital, University of Bern, Bern, Switzerland; 9 Division of Congenital and Structural Cardiology, University Hospitals Leuven, Leuven, Belgium; 10 KU Leuven Department of Cardiovascular Sciences, KU Leuven – University of Leuven, Leuven, Belgium; 11 Department of Adult Congenital Heart Disease, Chiba Cardiovascular Center, Chiba, Japan; 12 Department of Cardiology, Academic Medical Center, Amsterdam, the Netherlands; 13 National Taiwan University Hospital and Medical College, National Taiwan University, Taipei, Taiwan; 14 Center for Biobehavioral Health, Nationwide Children’s Hospital, Columbus, OH USA; 15 Adult Congenital Heart Center, Montreal Heart Institute, Université de Montréal, Montreal, Canada; 16 Adult Congenital Heart Disease Center, Helen DeVos Children’s Hospital, Grand Rapids, MI USA; 17 Pediatric Cardiology, Frontier Lifeline Hospital (Dr. K. M. Cherian Heart Foundation), Chennai, India; 18 Division of Cardiology, Hospital de Niños, Córdoba, Argentina; 19 Adult Congenital Heart Disease Center, Oslo University Hospital – Rikshospitalet, Oslo, Norway; 20 Centre for Person-Centred Care (GPCC), University of Gothenburg, Gothenburg, Sweden; 21 Adult Congenital Heart Unit, Sahlgrenska University Hospital/Östra, Gothenburg, Sweden; 22 Institute of Medicine, The Sahlgrenska Academy at University of Gothenburg, Gothenburg, Sweden; 23 Department of Health Sciences, University West, Trollhättan, Sweden; 24 Department of Public Health and Clinical Medicine, Umeå University, Umeå, Sweden; 25 Division of Cardiology, Stollery Children’s Hospital, University of Alberta, Edmonton, Canada; 26 Monash Heart, Monash Medical Centre, Monash University, Melbourne, Australia; 27 Department of Cardiology, Mater Dei Hospital, Birkirkara Bypass, Msida, Malta; 28 Adult Congenital Heart Disease Center, Cincinnati Children’s Hospital Medical Center, Cincinnati, OH USA; 29 Department of Congenital Heart Disease, Louis Pradel Hospital, Hospices civils de Lyon, Lyon, France; 30 Adult Congenital Heart Disease Program at Stanford, Lucile Packard Children’s Hospital and Stanford Health Care, Palo Alto, CA USA; 31 Adult Congenital Heart Disease Center, Washington University and Barnes Jewish Heart & Vascular Center, University of Missouri, Saint Louis, MO USA; 32 Clinical Psychology Service, IRCCS Policlinico San Donato, Milan, Italy; 33 Department of Biomedical Sciences for Health, Università degli Studi di Milano, Milan, Italy; 34 Adult Congenital Heart Disease Center University of Nebraska Medical Center/ Children’s Hospital and Medical Center, Omaha, NE USA; 35 Institute of Health and Care Sciences, University of Gothenburg, Gothenburg, Sweden; 36 Department of Paediatrics and Child Health, University of Cape Town, Cape Town, South Africa

**Keywords:** Congenital Heart Defects, Health Resources, Healthcare workforce, Patient Reported Outcome Measures, Quality of life, Staffing

## Abstract

**Background:**

The relationship between healthcare system inputs (e.g., human resources and infrastructure) and mortality has been extensively studied. However, the association between healthcare system inputs and patient-reported outcomes remains unclear. Hence, we explored the predictive value of human resources and infrastructures of the countries’ healthcare system on patient-reported outcomes in adults with congenital heart disease.

**Methods:**

This cross-sectional study included 3588 patients with congenital heart disease (median age = 31y; IQR = 16.0; 52% women; 26% simple, 49% moderate, and 25% complex defects) from 15 countries. The following patient-reported outcomes were measured: perceived physical and mental health, psychological distress, health behaviors, and quality of life. The assessed inputs of the healthcare system were: (i) human resources (i.e., density of physicians and nurses, both per 1000 people) and (ii) infrastructure (i.e., density of hospital beds per 10,000 people). Univariable, multivariable, and sensitivity analyses using general linear mixed models were conducted, adjusting for patient-specific variables and unmeasured country differences.

**Results:**

Sensitivity analyses showed that higher density of physicians was significantly associated with better self-reported physical and mental health, less psychological distress, and better quality of life. A greater number of nurses was significantly associated with better self-reported physical health, less psychological distress, and less risky health behavior. No associations between a higher density of hospital beds and patient-reported outcomes were observed.

**Conclusions:**

This explorative study suggests that density of human resources for health, measured on country level, are associated with patient-reported outcomes in adults with congenital heart disease. More research needs to be conducted before firm conclusions about the relationships observed can be drawn.

**Trial registration:**

ClinicalTrials.gov: NCT02150603. Registered 30 May 2014,

## Background

Healthcare expenditures have never been higher and medical costs are expected to continue to increase in all parts of the world [1]. In 2013, 7.8 trillion US $ were spent on healthcare worldwide, and this number is expected to increase up to 18.3 trillion US $ by 2040 [1]. This strong increase can be attributed to an ageing population and medical innovation [1]. Hence, more than ever, it is important to critically evaluate financial investments in health care and appraise the importance of ‘inputs’, such as human resources and the infrastructure of the healthcare system. Human resources of the healthcare system are *“all people engaged in actions whose primary intent is to enhance health”* [2]. Healthcare system infrastructure refers to *“formal and enduring structures that support health”,* such as hospitals and the number of available hospital beds [3].

In the late 1960s, a behavioral model of healthcare services use was developed by Andersen and colleagues to facilitate an understanding of which factors influenced patients’ use of healthcare services [4]. This model has expanded with the growth of supporting empirical evidence [5]. In the latest versions of the model (see eFig. 1), healthcare system inputs are considered part of the organization of the healthcare system and are included as contextual characteristics driving health consumption by patients [5]. The model assumes that contextual characteristics are both directly and indirectly associated with patient outcomes and that these relationships can be bidirectional.

Prior studies have investigated the role of human resources and infrastructure for health in predicting patient outcomes [6–9]. Physician and nurse staffing ratios and the availability of hospital beds per population have been associated with various outcomes including mortality, stage of diagnosis, prognosis, and quality of care [7–10]. However, minimal attention has been devoted to the associations between the human resources and the infrastructure, and outcomes which are particularly relevant from the patients’ perspective, namely patient-reported outcomes (PROs) [11–15]. Yet, in light of the current priority on patient-centered, holistic, and comprehensive care, the patient perspective should surely be incorporated in healthcare system evaluations [16]. Therefore, the relationship between country-level healthcare system inputs and PROs warrants investigation in public health and health services research. PROs are *“measurements based on a report that comes directly from the patient about the status of a patient’s health condition, without amendment or interpretation of the patient’s response by a clinician or anyone else”* [17]. Examples of PROs are self-reported health status or quality of life, these outcomes are included as such in the Andersen behavioral model (see eFig. 1) [5]. One study has investigated the association between the density of healthcare workforce (on country-level) and psychosomatic and mental health symptoms (on individual-level), but found no robust relationship [15]. As the Andersen behavioral model presumes a relationship between country-level healthcare system inputs and PROs, such investigations are warranted.

For the present study, we use data of adults with congenital heart disease (CHD). With a birth prevalence up to 9.3 per 1000 newborns, CHD is the commonest birth defect in neonates [18]. This population is of great interest to investigate the association between healthcare system inputs and PROs, for several reasons. First, in Western countries, due to significant medical advances in recent decades, over 90% of children with CHD now survive into adulthood, thus yielding an exponential growth of the population of adults with CHD [19]. Hence, this population is putting an increased burden on the healthcare system, with exponentially increasing healthcare utilization and costs [20]. From this perspective, CHD can be seen as an exemplar for life-cycle diseases. Second, CHD comprises a range of mild, moderate and complex heart defects. This variability in complexity reflects the broader spectrum of chronic diseases which also differ in complexity and severity. Third, as in other chronic conditions, a comprehensive understanding of adult CHD extends beyond medical outcomes and includes PROs. Inter-country variations in PROs among adults with CHD have previously been observed [13]. It is possible that certain healthcare system characteristics at the national level may play a role in this matter. Indeed, among adults with CHD, total healthcare expenditures per capita and healthcare system performance have been associated with PROs [13, 21]. The explained variance of these factors, however, ranged between 0.001 and 0.25%, suggesting that other aspects of the organization of the healthcare system require scrutiny. When exploring the relationship between healthcare system inputs and PROs in this heterogeneous chronic population, we can obtain valuable information that can be transferred, at least to a certain extent, to other chronic patient populations.

We aimed to test the presumed relationship between the inputs of the overall healthcare system and PROs of the Andersen behavioral model and to obtain a better understanding of the mechanism of inter-country differences in PROs. To achieve this aim, we investigated whether human resources and infrastructure for health on country level, operationalized as physician density, nurse density, and hospital bed density, were predictors of PROs in a large international sample of adults with CHD.

## Methods

### Study population and procedure

The present study was part of an overarching project entitled ‘Assessment of Patterns of Patient-Reported Outcomes in Adults with Congenital Heart disease – International Study’ (APPROACH-IS). A total of 4028 adults with CHD from 15 countries, comprising five continents, were included in this project [13, 22, 23].

Inclusion criteria were: (i) diagnosis of CHD, defined as *“a structural abnormality of the heart and/or intra-thoracic great vessels that is present at birth and of actual or potential functional significance*” [24]; (ii) aged 18 years or older; (iii) diagnosis established before the age of 10 years; (iv) continued follow-up at a CHD center or included in a national/regional registry; and (v) possessing physical, cognitive, and language capabilities required to complete self-reported questionnaires. Exclusion criteria were: prior heart transplantation and primary pulmonary hypertension [22]. Data collection was carried out from April 2013 through March 2015. In a previous paper, rationale, design, and methods of APPROACH-IS have been detailed [22]. The study protocol was registered at ClinicalTrials.gov: NCT02150603.

### Measures (individual-level data)

For the present study, data on five PROs were analyzed: perceived physical health and mental health status (12-item Short Form Health Survey), psychological distress (Hospital Anxiety and Depression Scale), health behavior (Health Behavior Scale–Congenital Heart Disease), and quality of life (Linear Analog Scale). Further details on the used set of questionnaires have been reported previously [22] and can be found online in supplementary material (see eTable 1).

### Healthcare system inputs (country-level data)

In order to capture inputs of the healthcare system, human resources and infrastructure measurements were assessed. Country-level human resources for health were operationalized in terms of physician density as well as density of nurses. Physician density per 1000 people was defined as the number of physicians, including generalists and specialist medical practitioners, per 1000 citizens in the country’s population [25, 26]. Nurse density per 1000 people was operationalized as the number of professional nurses, professional midwives, auxiliary nurses, auxiliary midwives, enrolled nurses, enrolled midwives and other associated personnel, such as dental nurses and primary care nurses, per 1000 people within a country’s population [26]. Country-level health infrastructure was expressed as the hospital bed density per 10,000 population, which included the total number of hospital beds excluding labour and delivery beds per 10,000 population [26]. These data were obtained from the World Bank and the World Health Organization (WHO) [25, 26]. Since data from Taiwan are lacking in the WHO repository, the Taiwan Statistical Data Book was consulted to obtain the required data for Taiwan [27]. For most countries, data from 2015 have been selected. If this score was unavailable, the nearest-to-2015 score has been selected (see eTable 2).

### Statistical analyses

Continuous data are presented as medians and interquartile ranges. Categorical variables are presented as absolute numbers and percentages.

The association of healthcare system inputs and PROs was estimated through general linear mixed models (GLMM). GLMM are an extension to the generalized linear model (GLM) with random effects in addition to the usual fixed effects. In the analyses, a 2-level hierarchical structure was taken into account, in which patients were nested within countries. Thereby, random effects were included at the country-level and the model corrects for differences between countries, so called ‘unmeasured country differences’. We modeled five PROs as dependent variable with the healthcare system inputs as predictors. Only patients for whom full data were available for all variables of interest (*n* = 3588) were included, as only a relatively small proportion of patients had missing data for PROs (0.05–1.89%). Therefore, it was unnecessary to perform multiple imputation. The GLMM analyses were performed in three steps:

First, separate univariable general linear mixed models were applied to estimate the associations between density of physicians, density of nurses and density of hospital beds and the five PROS, being physical health status, mental health status, psychological distress, health behavior, and quality of life.

Second, multivariable general linear mixed models were performed for the three health care system inputs and the five PROs, with adjustments for patient-specific variables that previously have been found to predict PROs (i.e., sex, age, employment status, marital status, highest educational level, complexity of the health disease and the patient’s functional ability (New York Heart Association classification) [13]) and unmeasured country differences.

Third, in order to estimate the robustness of the results, we performed sensitivity analyses in which we excluded countries with an outlying density of more than two standard deviations from the mean. The multivariable general linear mixed models were repeated while excluding these centers.

Data analysis was performed using IBM SPSS Statistics for Windows, version 24 (Armonk, NY: IBM Corp.). Tests were two-sided and a *p* < 0.05 was considered as statistical significant.

## Results

### Sample characteristics

In all, 3588 adults with CHD from 15 countries were included in the analyses. The country with the greatest number of included patients was the USA (666; 18.6%). Most patients had moderate disease complexity (1740; 48.5%), were women (1876; 52.3%) and reported no limitations during physical activity (NYHA Class 1) (1951; 54.4%). Detailed information on the demographic and clinical characteristics of the sample is given in Table 1.

**Table 1:** Demographic and clinical characteristics of the sample (*n* = 3588)

Variables	n (%)
Sex: female	1876 (52.3)
Median age in years	31.0 (IQR: 16.0)
Educational level	
Less than high school	176 (4.9)
High school	1508 (42.0)
College degree	777 (21.7)
University degree	1127 (31.4)
Employment status	
Part-time or full-time work	2343 (65.3)
Job seeking, unemployed, or disabled	424 (11.8)
Homemaker or retired	268 (7.5)
Full-time student	296 (8.2)
Other	257 (7.2)
Marital status	
Married or living with partner	1831 (51.0)
Never married	1582 (44.1)
Divorced or widowed	172 (4.8)
Other	3 (0.1)
Children: yes	1407 (39.2)
Patient-reported functional ability (New York Heart Association)	
Asymptomatic (Class I)	1951 (54.4)
Slight limitation (Class II)	1255 (35.0)
Marked limitation (Class III)	254 (7.1)
Symptoms at rest (Class IV)	128 (3.5)
Complexity of heart defect	
Simple	936 (26.1)
Moderate	1740 (48.5)
Complex	912 (25.4)
Country	
Argentina	145 (4.0)
Australia	125 (3.5)
Belgium	256 (7.1)
Canada	462 (12.9)
France	83 (2.3)
India	184 (5.1)
Italy	49 (1.4)
Japan	235 (6.6)
Malta	100 (2.8)
Norway	162 (4.5)
Sweden	425 (11.8)
Switzerland	219 (6.1)
Taiwan	248 (6.9)
the Netherlands	229 (6.4)
USA	666 (18.6)

Note. IQR = interquartile range

### Healthcare system inputs

**Fig. 1:**
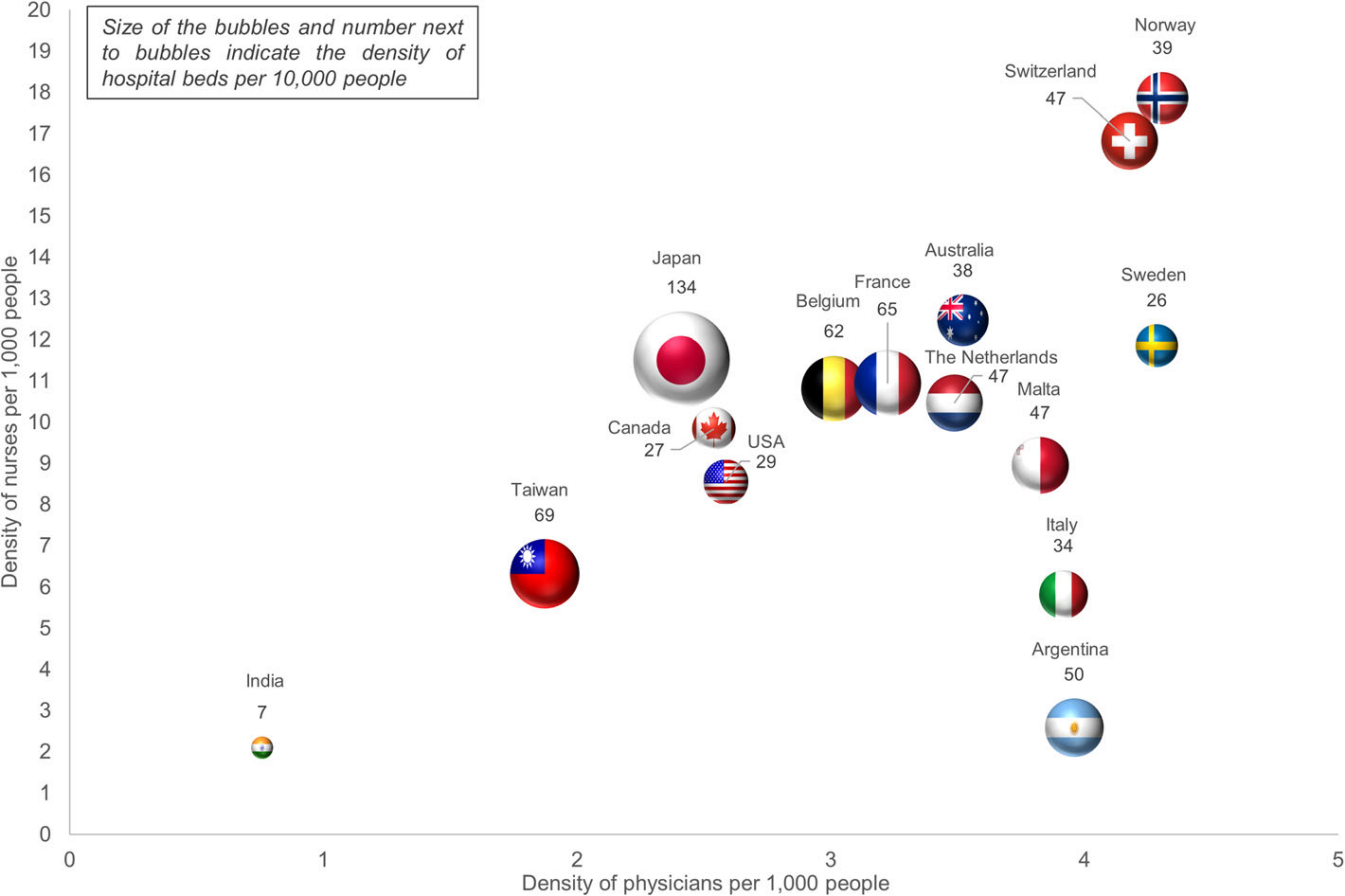
Human resources and infrastructure of the healthcare system of countries included in APPROACH-IS**.** Size of the bubbles and number next to bubbles indicate the density of hospital beds per 10,000 people; Sources: World Bank [25], World Health Organization [26] and Taiwan Statistical Data Book 2016 [27]

In Figure 1, we summarize country-level healthcare system inputs data from the different participating countries. Density of physicians was highest in Norway (4.4 per 1000 people) and lowest in India (0.8 per 1000 people). Norway had the highest density of nurses (17.9 per 1000 people), whereas India had the lowest (2.1 per 1000 people). With 134 beds per 10,000 people, Japan had the highest density of hospital beds, whereas India had the lowest density (7 beds per 10,000 people). For all countries, the mean density of physicians was 3.2 per 1000 people (SD: 1.0), the mean density of nurses was 9.8 per 1000 people (SD: 4.4) and the mean density of hospital beds was 48.1 per 10,000 people (SD: 28.9). Hence, two outlying values were noticed, being the density of physicians in India and the density of hospital beds in Japan. In eTable 2 of the supplementary material, a more detailed overview of these data, the year these data were collected, and a brief description of the overall healthcare system, can be found.

### Patient-reported outcomes

PROs of this sample are described in eFig. 2 and have been reported and discussed in more detail in a prior publication [13]. Patients from Malta had the best self-reported physical and mental health, whereas patients from India and France had the worst self-reported physical and mental health, respectively. The lowest scores of psychological distress (i.e., depressive and anxiety symptoms) were found in patients living in the Netherlands, whereas the highest scores were reported by patients from India. Moreover, Indian patients demonstrated higher prevalence of health risk behaviors (i.e., smoking, drinking, drugs, lack of physical activity, and problematic oral care), whereas Swedish patients demonstrated the lowest prevalence of health risk behaviors. Patients from Switzerland demonstrated the best quality of life, and Japanese patients the worst.

### Association between healthcare system inputs and PRO

In the univariable general linear mixed models, higher physician density was associated with better self-reported physical and mental health, less psychological distress, and better quality of life (Table 2). A higher number of nurses per population was associated with better physical health status and less risky health behavior. A high hospital bed density was associated with lower quality of life.

**Table 2:** General Linear Mixed Models with healthcare system inputs as predictors of PROs (*n* = 3588)

	Physical health status	Mental health status	Psychological distress	Total health risk score	Quality of life
**Univariable analyses**					
Density physicians	3.16 (0.78) **	1.90 (0.71) *	−0.84 (0.20) **	−2.31 (1.30)	1.59 (0.69) *
Density nurses and midwifes	0.50 (0.23) *	0.22 (0.19)	−0.13 (0.06)	−0.73 (0.27) *	0.07 (0.18)
Density beds	0.03 (0.04)	−0.01 (0.03)	−0.0008 (0.01)	0.04 (0.05)	−0.06 (0.02) *
**Multivariable analyses**					
Density physicians	3.11 (0.84) **	1.51 (0.59) *	−0.72 (0.20) **	−2.41 (1.26)	1.33 (0.69)
Density nurses and midwifes	0.51 (0.23) *	0.15 (0.16)	−0.12 (0.05) *	−0.77 (0.25) **	0.05 (0.18)
Density beds	0.05 (0.04)	0.002 (0.02)	−0.002 (0.01)	0.04 (0.05)	−0.06 (0.02) *
**Sensitivity analyses**					
Density physicians *(without India)*	2.54 (1.14) *	2.00 (0.77) *	−0.79 (0.27) *	−1.31 (1.66)	2.09 (0.90) *
Density beds *(without Japan)*	−0.01 (0.07)	−0.04 (0.04)	− 0.008 (0.02)	−0.006 (0.09)	− 0.02 (0.04)

Values in the table are Estimates (Standard Error) of the General Linear Mixed Models; * refers to significance of estimate (see below); Multivariable analyses are adjusted for patient characteristics (sex, age, employment status, marital status, highest educational level, complexity of the health disease and the patient’s functional ability) and unmeasured country differences (since country has been seen as random effect in the analyses). Physical and mental health status: higher scores reflect better perceived health; Psychological distress: higher scores reflect more symptoms of depression and anxiety; Total health risk score: higher scores reflect unhealthier behaviour; Quality of life: higher scores reflect higher quality of life; Density of physicians per 1000 people; Density of nurses per 1000 people; Density of beds per 10,000 people

* < 0.05, ** < 0.01, *** < 0.001

Multivariable general linear mixed models, adjusted for patient characteristics and unmeasured country differences, showed very similar results (Table 2). However, the association between density of physicians and quality of life was no longer significant and the association between density of nurses and psychological distress turned significant in the multivariable analyses.

Because the density of physicians in India and the density of hospital beds in Japan deviated more than two standard deviations from the mean in the sample, we performed sensitivity analyses excluding these countries on an individual basis (Table 2). After removing India, higher density of physicians remained associated with better self-reported physical and mental health status, less psychological distress, and better quality of life. When Japan was excluded from the analyses, no associations between bed density and PROs were observed.

## Discussion

We explored the relationship between healthcare system inputs and PROs, as proposed in the Andersen behavioral model. The results of the present study demonstrated that country-level density of physicians and nurses were associated with PROs among adults with CHD. Although replication in future studies is needed, our results suggested that human resources for health might directly or indirectly affect PROs and might partly explain the inter-country variations in PROs reported previously [13].

### Physician density

In the current study, univariable, multivariable, and sensitivity analyses indicated that a higher country-level physician density was associated with favourable PROs in adults with CHD. More precisely, we found that a higher number of physicians was associated with better self-reported health, less psychological distress, and better quality of life. This is not in line with the study of Riehm and colleagues, in which no association between the density of physicians and psychiatrists (on country-level) and better mental health of adolescents was found [15]. However, most previous research, which investigated the relationship between the density of physicians and outcomes at a national level, both patient-reported and other patient outcomes, are in line with our findings. In Canada, the variation of physician density within the country determined self-reported general health status [12]. It was estimated that adding an additional family physician per 10,000 population would yield a 2 to 4% increase in self-reported general health [12]. Similarly, an American study demonstrated that a higher supply of primary care physicians was associated with a higher probability of reporting good self-rated health [28]. In addition, a study from Hungary showed that more physicians per 100,000 inhabitants was associated with better recognition of depression and a lower suicide rate [29]. It can be hypothesized that earlier diagnosis and timely treatment of health problems may explain the associations between higher physician density with better health status and less psychological distress. Indeed, greater physician density has been shown to be associated with lower mortality rates, earlier stage of diagnoses, and better prognosis [7–9]. As yet another example, the geographical variation in breast cancer mortality in the United States was shown to be partly explained by the number of physicians [8]. Our study suggested that improved outcomes do not only pertain to mortality and morbidity, but also to PROs.

### Nurse density

We also found that a higher country-level nurse density was associated with better perceived physical health, less psychological distress, and less risky health behaviors. A higher nurse density is likely to yield more absolute hours available for direct nursing care. A prior study in the United States showed that more hours of provision of direct nursing care on a shift was related to lower levels of pain and better self-reported health status of patients [30]. Hence, the time that a nurse has for direct patient care is probably a mediator for the association between nurse density and self-reported health, as it can be assumed that when the nurse density increases, the patient-to-nurse ratio decreases. Our finding that a higher density of nurses was associated with less psychological distress may initially seem to contradict those of an American study conducted in nursing homes, in which optimal staffing ratios were related to higher levels of depression [11]. However, this finding was determined to be due to the fact that higher nurse density led to improved recognition (i.e., diagnosis) of depression and anxiety [11]. This was confirmed in another study in which the predicted probability of a diagnosis of depression or anxiety increased from 14 to 16% when the ratio changed from over five patients per nurse to under three [31].

### Hospital bed density

Higher hospital bed density was associated with lower quality of life in uni- and multivariable analyses. However, the hospital bed capacity of Japan (i.e., 134 per 10,000 people) is by far the highest in the world [32], and is an outlier in our sample. In addition, we previously showed that Japanese patients enrolled in APPROACH-IS had the lowest average quality of life. We thus suspected that the association between bed density and quality of life could be fully explained by the outlying hospital bed capacity of Japan. This was confirmed by sensitivity analysis, which showed no associations between bed density and PROs after Japan was excluded. The number of hospital beds is an indicator for the capacity and availability of hospital infrastructure. But, it does not seem to be of great importance for patients with CHD. It could be that hospital beds are not the best indicator for this population, as most patients make use of the outpatient clinics. Therefore, in future studies in patients with CHD, it could be interesting to bring PROs of adults with CHD in association with the number of outpatient clinics or outpatient clinic visits.

### Methodological considerations

To date, most research on the association between health system inputs and PROs have been performed on a national level [8, 11, 12]. These studies investigated regional or institutional variance, but did not allow us to appraise variability between countries. Hence, the unique contribution of our study was the examination of healthcare system inputs (on country level) and PROs (on individual level) from an international perspective. Nonetheless, there are some methodological limitations to consider.

First, we used healthcare input data at country level and PROs at the level of individual patients, who were recruited from one or more centers in the respective countries. Although we performed multilevel analyses to control for unmeasured country differences and to take into account that patients are nested in countries, it would have been interesting to investigate human resources and PROs both measured at country or center level.

Second, although we included data from 15 countries around the globe, all but two (i.e., India and Argentina) were high income countries. To gain a richer understanding of the impact of healthcare system inputs at the international level, it will be important that future studies include more low- and middle-income countries. Furthermore, in most countries, there is regional variation in healthcare system inputs. Hence, it could be that the results have been influenced by the fact that some centers are located in high resources areas. Also this issue should be examined in future studies.

Third, although the Andersen behavioral model suggested both a direct and indirect link between contextual organization of the healthcare system and PROs (see eFigure 1) [5], the variables are distal from each other. Indeed, it is highly unlikely that the associations are direct. It can be assumed that other healthcare system characteristics (e.g., accessibility to care, including aspects of financing and insurance) or country specific characteristics (e.g., educational level or overall wealth of the population) might be confounding the association. The present study, therefore, warrants future research in which the underlying mechanisms and the intermediate variables are investigated thoroughly.

Fourth, the Andersen behavioral model assumes that the relations are bidirectional [5]. Because our study used an ecological cross-sectional design [22], we cannot draw conclusions in terms of the direction of effects, nor could causality be tested. Since this cross-sectional study provided strong indications about this association, subsequent studies should investigate the associations longitudinally by using multiple time points to confirm the presumed bidirectional association of the Andersen behavioural model.

Fifth, it is unknown whether our results pertaining to adults with CHD may be generalized to adults with other health conditions. As a sample case, CHD does represent a broad spectrum of mild, moderate, and complex chronic diagnoses. However, it would be interesting to investigate whether our observations could be replicated among patients with other chronic health conditions. Furthermore, there was no control group available for this study, in which the same patient-reported outcomes were measured using a similar methodology. But, it would be interesting in future studies to add a healthy control group or general population normative data to increase the generalizability and transferability of the findings.

In addition, this study focused on the density of physicians, nurses, and hospital beds. These are important indicators of the healthcare system, since they have been shown to be associated with the national performance of the system [33]. However, other aspects of the comprehensive organization of the healthcare system, such as the overall performance, accessibility to care and quality of care metrics should also be considered in future studies. Moreover, for the population of adults with CHD, it would be interesting to study the association between PROs and the density of human resources that is most relevant to them, namely the density of congenital cardiologists or specialized nurses. Additionally, it would be interesting to subsequently look at the combination of these professions and their impact on PROs, since they interact highly with each other and are both needed in the provision of good care.

Moreover, it is contradictory that midwives have been included in the nurse density and maternity beds have been excluded in the bed density. However, this is how the data were represented by the World Health Organization and the World Bank. Leaving out certain data for our analyses would have impacted the comparability and reliability of the data, since the available information on the number of midwives was incomplete or outdated for several countries.

Finally, valid and reliable self-reported instruments were used to measure PROs. Consequently, patients with cognitive impairment and learning difficulties were excluded in the study, which might also influence the generalizability of the findings. However, a study on the Swedish data of APPROACH-IS has shown that differences between participants and non-participants in demographic, clinical, and health status characteristics were small [34].

Understanding the influence of density of human resources and infrastructure on outcomes is an important consideration for health policy and planning [12]. Indeed, the study should be seen as a preliminary step for researchers to investigate the influence of healthcare system inputs on PROs. It is likely only with the availability of such scientific data that we will be able to advise policy makers about aspects of the healthcare system impacting PROs.

## Conclusion

This explorative study provides a first indication that country-level density of healthcare system inputs are associated with PROs in adults with CHD. More specifically, higher physician density was associated with better self-reported physical and mental health, less psychological distress, and better quality of life. Higher density of nurses was associated with better physical health status, less psychological distress, and less risky health behaviors. Density of hospital beds was not associated with PROs. Before firm conclusions about the relationships observed can be drawn and before recommendations for policy-making can be made, more research ought to be conducted in order to replicate the present findings and to scrutinize the mediating mechanisms between characteristics of the healthcare system and outcomes as perceived by the patients.

## Supplementary information

**Additional file 1: Table S1.** Overview of variables and measurements. **Table S2.** Detailed overview of the healthcare system inputs and other healthcare system characteristics of countries included in APPROACH-IS. **Figure S1.** Andersen behavioral model of health services use, sixth revision (Reproduced from [13]). Permission to reuse figure was obtained from John Wiley and Sons. **Figure S2.** Mean and standard deviation of patient-reported outcomes in 3588 adults with congenital heart disease.

## Data Availability

The datasets generated and/or analysed during the current study are available from the corresponding author on reasonable request.

## Abbreviations

APPROACH-IS: Assessment of Patterns of Patient-Reported Outcomes in Adults with Congenital Heart disease – International Study
CHD: Congenital Heart Disease
GLM: Generalized Linear Model
GLMM: General Linear Mixed Models
IQR: Interquartile Range
NYHA: New York Heart Association Classification
PRO: Patient Reported Outcomes
SD: Standard Deviation
WHO: World Health Organization
