# Supplementary material for: Healthcare system inputs and patient-reported outcomes: a study in adults with congenital heart defect from 15 countries
Source: BMC Health Serv Res. 2020 Jun 3;20:496. doi: 10.1186/s12913-020-05361-9 (PMC7268498; doi:10.1186/s12913-020-05361-9)
Supplement: Supplementary file 1 — Additional file 1: Table S1. Overview of variables and measurements. Table S2. Detailed overview of the healthcare system inputs and other healthcare system characteristics of countries included in APPROACH-IS. Figure S1. Andersen behavioral model of health services use, sixth revision (Reproduced from [13]). Permission to reuse figure was obtained from John Wiley and Sons. Figure S2. Mean and standard deviation of patient-reported outcomes in 3588 adults with congenital heart disease. [file 12913_2020_5361_MOESM1_ESM.pdf]

Supplementary material

eTable 1. Overview of variables and measurements

**Variables**	**Definition used in this study**	**Measurement**	**Interpretation of measurement**
Patient-reported health status	The impact of a disease according to the patient, including symptoms, functional status, and health-related quality of life [1]	12- item Short- form Health Survey version 2 (SF-12) [2]	• Physical Component Summary (PCS) and Mental Component Summary (MCS) ranging from 0 to 100;<br>• Higher scores = better perceived health
Psychological distress	The level of internalizing, emotional distress, depressive and anxious symptoms	Hospital Anxiety and Depression Scale (HADS) [3]	• HADS scores ranging from 0 to 21;<br>• Higher scores = more symptoms<br>• Because HADS was found to be accurate when screening psychological distress, a general psychological distress score was used instead of using two subscores for depression and anxiety [20].
Health behaviors	Activities that a person undertakes to prevent disease or to improve health and well-being [4]	Health-Behavior Scale–Congenital Heart Disease (HBS-CHD) [5]	• Total health risk score ranging from 0 to 100;<br>• Higher scores = unhealthier behavior
Quality of life	The degree of overall life satisfaction that is positively or negatively influenced by individuals' perception of certain aspects of life important to them, including matters both related and unrelated to health [6]	Linear Analog Scale (LAS) [7]	• LAS score ranging from 0 (worst imaginable quality of life) to 100 (best imaginable quality of life);<br>• Higher scores = better quality of life

eTable 2. Detailed overview of the healthcare system inputs and other healthcare system characteristics of countries included in APPROACH-IS

**Healthcare system inputs** / **Countries**	Density of physicians (per 1,000 population)	Measured in (year)	Density of nurses (per 1,000 population)	Measured in (year)	Density of hospital beds (per 10,000 population)	Measured in (year)	Brief description of the healthcare system	
							Model of health system financing [8], [9]	Health expenditure as a % of the GDP in 2015
Argentina	3.96	2017	2.58	2017	50	2014	Universal public-private insurance system	6.8
Australia	3.5213	2015	12.4667	2015	38	2014	Universal government-funded health system	9.4
Belgium	3.0138	2015	10.8193	2015	62	2014	Universal public insurance system	10.5
Canada	2.5388	2015	9.8398	2015	27	2012	Universal government-funded health system	10.4
France	3.2237	2015	10.9403	2015	65	2013	Universal public insurance system	11.1
India	0.759	2016	2.0981	2016	7	2011	Non-universal insurance system	3.9
Italy	3.9174	2015	5.8184	2015	34	2012	Universal government-funded health system	9
Japan	2.412	2016	11.518	2015	134	2012	Universal public insurance system	10.9
Malta	3.826	2015	8.95	2015	47	2014	Universal government-funded health system	9.6
Netherlands	3.4875	2015	10.4675	2015	47	2009	Universal private health insurance system	10.7
Norway	4.394	2015	17.8622	2015	39	2013	Universal government-funded health system	10
Sweden	4.2861	2015	11.8503	2015	26	2013	Universal government-funded health system	11
Switzerland	4.1782	2015	16.8152	2015	47	2013	Universal private health insurance system	12.1
Taiwan	1.873	2015	6.316	2015	69	2015	Universal government-funded health system	5.9
USA	2.5858	2015	8.55	2015	29	2013	Non-universal insurance system	16.8

Sources. World Bank Open Data [10], World Health Organization [11] and Taiwan Statistical Data Book 2016 [12]

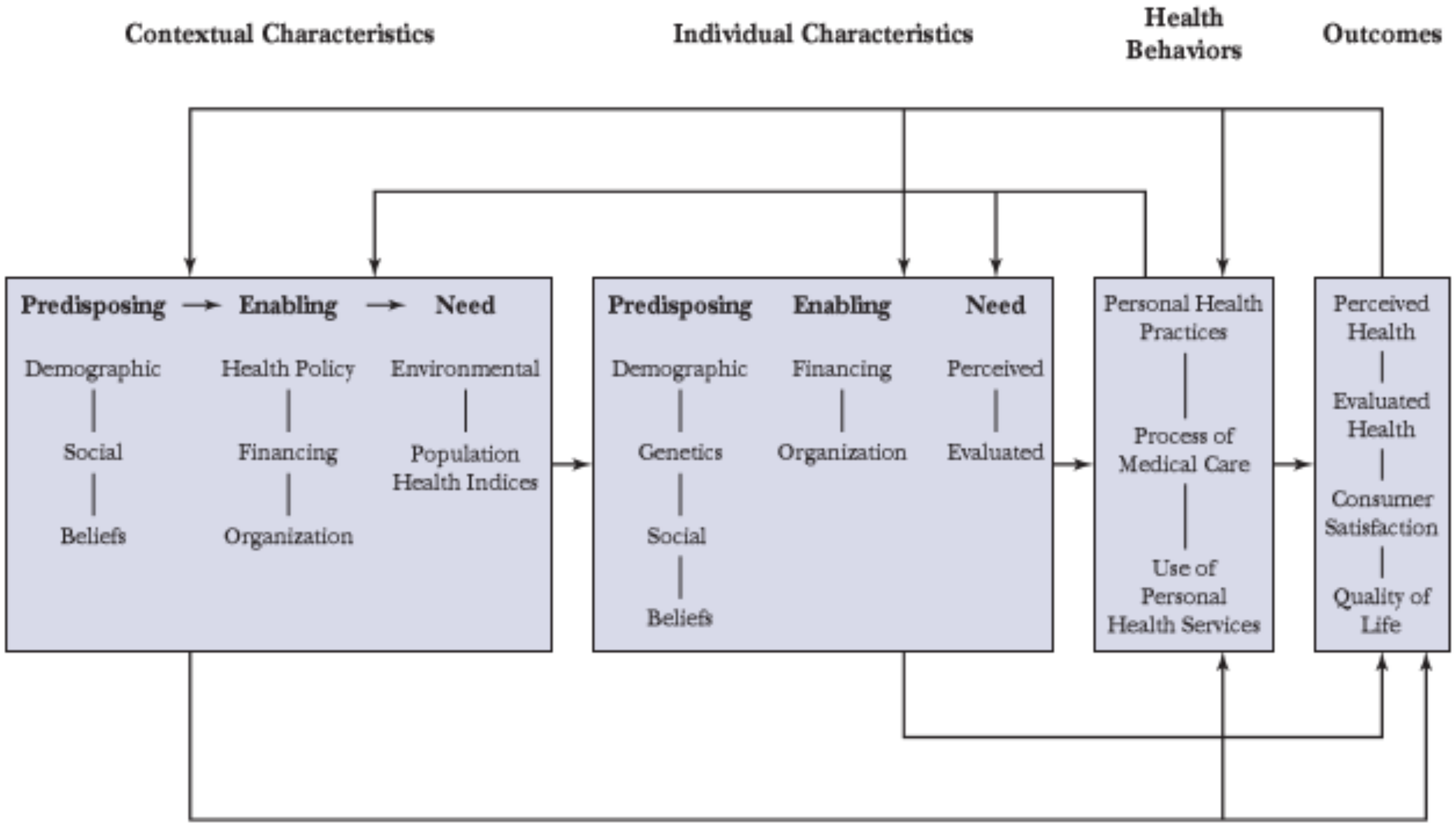

eFigure 1. Andersen behavioral model of health services use, sixth revision (Reproduced from [13]). Permission to reuse figure was obtained from John Wiley and Sons.

Countries	Physical health status (0-100)	Mental health status (0-100)	Psychological distress (0-42)	Total health risk score (0-100)	Quality of life (0-100)
Argentina (n=145)	79.3 (18.2)	72.1 (18.5)	8.8 (6.0)	28.4 (16.4)	80.0 (14.5)
Australia (n=125)	76.7 (19.2)	71.2 (18.3)	8.1 (6.2)	26.0 (16.4)	82.5 (14.2)
Belgium (n=256)	78.9 (19.5)	74.5 (17.9)	8.5 (6.4)	23.0 (16.4)	77.7 (13.4)
Canada (n=462)	79.1 (17.8)	69.8 (16.8)	10.2 (5.3)	18.8 (17.2)	76.0 (15.7)
France (n=83)	73.4 (22.5)	66.4 (20.7)	10.6 (6.7)	30.2 (16.3)	78.2 (15.0)
India (n=184)	66.9 (20.6)	68.6 (19.8)	10.7 (6.5)	34.8 (13.2)	76.3 (17.8)
Italy (n=49)	79.5 (17.8)	76.9 (17.3)	7.5 (7.0)	25.4 (16.4)	80.9 (15.1)
Japan (n=235)	81.3 (17.6)	72.8 (19.3)	9.1 (8.0)	30.1 (16.7)	70.7 (21.6)
Malta (n=100)	85.8 (15.8)	77.2 (17.1)	8.0 (6.6)	32.8 (16.3)	81.2 (13.3)
Norway (n=162)	77.8 (20.5)	70.1 (20.0)	7.7 (6.2)	18.5 (18.2)	76.9 (17.3)
Sweden (n=425)	84.3 (19.9)	76.8 (19.5)	7.6 (6.5)	16.8 (16.0)	79.6 (17.4)
Switzerland (n=219)	82.6 (19.0)	76.4 (16.6)	7.1 (5.9)	17.9 (16.3)	83.1 (14.2)
Taiwan (n=248)	71.1 (18.3)	67.0 (16.9)	9.1 (5.7)	22.0 (15.2)	75.5 (14.1)
the Netherlands (n=229)	78.1 (18.9)	76.0 (17.0)	6.8 (5.1)	19.2 (16.9)	80.2 (12.1)
USA (n=666)	75.6 (23.1)	71.9 (20.5)	9.3 (6.8)	21.7 (16.5)	80.6 (17.0)
**All countries (n=3588)**	**78.1 (20.3)**	**72.5 (18.8)**	**8.8 (6.4)**	**22.5 (17.1)**	**78.4 (16.3)**

(Column header group label: **Patient-reported outcomes**)

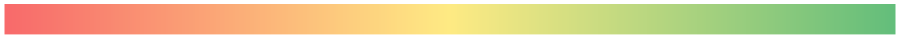

eFigure 2. Mean and standard deviation of patient-reported outcomes in 3588 adults with congenital heart disease

References

1. Rumsfeld JS, Alexander KP, Goff DC, Jr., et al. Cardiovascular health: the importance of measuring patient-reported health status: a scientific statement from the American Heart Association. Circulation. 2013;127(22):2233-49.
2. Ware JE, Kosinski M, Turner-Bowker DM, Sundaram M, Gandek B, Maruish ME. User's Manual for the SF-12v2 Health Survey Second Edition. QualityMetric, Incorporated; 2009.
3. Zigmond AS, Snaith RP. The hospital anxiety and depression scale. Acta Psychiatr Scand. 1983;67(6):361-70.
4. Conner M, Norman P. Predicting health behaviour: Research and practice with social cognition models. Second edition ed. Maidenhead, England: Open University Press; 2005.
5. Goossens E, Luyckx K, Mommen N, et al. Health risk behaviors in adolescents and emerging adults with congenital heart disease: psychometric properties of the Health Behavior Scale-Congenital Heart Disease. Eur J Cardiovasc Nurs. 2013;12(6):544-57.
6. Moons P, Van Deyk K, Marquet K, et al. Individual quality of life in adults with congenital heart disease: a paradigm shift. Eur Heart J. 2005;26(3):298-307.
7. Moons P, Van Deyk K, De Bleser L, et al. Quality of life and health status in adults with congenital heart disease: a direct comparison with healthy counterparts. Eur J Cardiovasc Prev Rehabil. 2006;13(3):407-13.
8. Kulesher RR, Forrestal EE. International models of health systems financing. J Hosp Adm. 2014;3(4):127.
9. International Social Security Association. Social Security Programs Throughout the World. https://www.ssa.gov/policy/docs/progdesc/ssptw/. Accessed May 2020.

10. World Bank. World Bank Open Data. https://data.worldbank.org/. Accessed June 2019.

11. World Health Organization. Global Health Observatory data repository. Available at: http://apps.who.int/gho/data/node.main.A1444 and http://apps.who.int/gho/data/view.main.HS07v. Accessed June 2019.

12. National Development Council R.O.C. (Taiwan). Taiwan Statistical Data Book 2016.

13. Andersen RM, Davidson PL, Baumeister SE. Improving access to care in America. In: Kominski EF, ed. Changing the U.S. health care system: key issues in health services, policy and management. 4th edition. San Francisco: Jossey-Bass; 2013:33-69.
